# The anti-Müllerian hormone gene's second exon is associated with the reproductive performance of Jinghai Yellow chickens

**DOI:** 10.5194/aab-64-45-2021

**Published:** 2021-02-05

**Authors:** Yulin Wu, Manman Shen, Xuemei Yin, Yanjun Duan, Shanshan Zhang, Hao Ding, Lan Chen, Tao Zhang, Genxi Zhang, Jinyu Wang

**Affiliations:** 1 Jiangsu Key Laboratory of Animal Genetic Breeding and Molecular Design, College of Animal Science and Technology, Yangzhou University, Yangzhou, China; 2 College of Biotechnology, Jiangsu University of Science and Technology, Zhenjiang, China

## Abstract

Anti-Müllerian hormone (AMH), a member of the transforming growth factor-β superfamily, plays important regulatory roles in follicular development and sex differentiation. Although much has been learned about the impact of polymorphisms of *AMH* on reproduction in animals, the effect on chicken reproduction is not well explored. In this study, the polymorphism of five exons of *AMH* gene and its effect on the reproductive performance of Jinghai Yellow chickens were studied. Primers for the amplification of *AMH* exons were designed, and Sanger sequencing was performed. Finally, only the polymorphism in the second exon of the *AMH* gene was found in the present population. Polymorphisms in the second exon of the *AMH* gene in 246 Jinghai Yellow hens and their associations with reproductive traits were analyzed. In total, four single nucleotide polymorphism (SNP) mutations were detected in the second exon of the *AMH* gene: g.1868A>C (AA, aa and Aa); g.1883G>A (BB, bb and Bb); g.1987G>A (CC, cc and Cc); and g.1996A>G (DD, dd and Dd). Only the mutation of g.1996A>G affected the reproductive traits: the age of laying first egg (AFE) of dd genotype was significantly (p<0.01) earlier than that in the DD and Dd hens. Moreover, the egg number by 300 d old (EN300) of dd individuals was significantly higher than that of DD and Dd individuals (p<0.01). Thus, we inferred that the dd genotype is the beneficial genotype. Additionally, AFE and EN300 showed significantly better performance in both the H2H2 and H7H7 diplotypes compared with other diplotype individuals (p<0.01). Thus, the H2H2 and H7H7 genotype had the best combination of AFE and EN300. Our study may allow for molecular marker section in poultry breeding.

5 February 2021

## 1 Introduction

Anti-Müllerian hormone (AMH) is a member of the transforming growth factor (TGF-β) superfamily and plays important roles in follicular development and gender differentiation (Cutting et al., 2014). AMH is a dimer glycoprotein with a molecular weight of 140 kDa, which consists of two 70 kDa subunits linked by disulfide bonds. The human *AMH* gene is located on the short arm of chromosome 19 and has a length of 2.4–2.8 kb. It contains five exons and encodes a 560-amino-acid protein precursor (Rey et al., 2003). However, in original red domestic chickens, the *AMH* gene is 4.0 kb, has five exons and is located on chromosome 28. The encoded *AMH* protein contains 644 amino acid residues (Li, 2017). In 1999, Durlinger et al. discovered that the number of primordial follicles in mice lacking *AMH* is more than in normal mice. They determined that *AMH* inhibits the promotive action of follicle-stimulating hormone on follicular growth and development (Durlinger et al., 1999). Visser et al. (2007) corroborated these findings by observing wild and *AMH* knockout mice, which showed that there were significant differences in the number of small preantral and large preantral follicles in *AMH* null mice. Follicles in the ovaries of mice lacking *AMH* were depleted in advance (Durlinger et al., 1999), which indicated that follicles were recruited in large quantities without the inhibition of *AMH*, leading to a decline in the ovarian reserve function.

At present, research on AMH in human reproductive diseases mainly focuses on premature ovarian failure and polycystic syndrome (Zhang et al., 2013). In animal husbandry, the concentration of serum AMH is mainly used to determine the superovulation effects on female livestock (Li et al., 2018). In mice, AMH pretreatments before superovulation effectively increase the ovulation rate (Hayes et al., 2016). Shen et al. (2017) found a significant single nucleotide polymorphism (SNP) association with chicken follicle numbers using a whole genome analysis study. In humans, *AMH* gene variations are used as prognostic biomarkers of follicular development. Using a transcriptome analysis of hen ovaries, Zhang et al. (2019) showed that *AMH* is a candidate gene responsible for egg-laying traits. Here, polymorphisms in the five exons of the *AMH* gene and their different alleles' correlations with reproductive performance were analyzed in Jinghai Yellow chickens. The results reveal the influences of *AMH* gene mutations on the reproductive performance of Jinghai Yellow chickens.

## 2 Materials and methods

### 2.1 Ethics statement

The study protocol was approved by the Animal Care Committee of the Department of Animal Science and Technology of Yangzhou University and conducted in accordance with the guidelines of the Animal Use Committee of the Chinese Ministry of Agriculture. All efforts were made to minimize animal suffering.

### 2.2 Test materials

The test chickens came from the Jiangsu Jinghai Poultry Industry Group Co., Ltd. In total, 246 hens from the 13th generation of the core group of Jinghai Yellow chickens were randomly selected. The Jinghai Yellow chicken, which is a breed of Chinese yellow-feathered broiler, is very popular with Chinese consumers. All hens were reared under natural light and were transferred to single cages from 16 weeks old; the animals had a 10 % feed restriction and free access to water, according to the feeding standards. Light was increased by 1 h per week until 16 h of light was provided. Statistics related to reproductive performance included age at first egg (AFE), body weight at first egg (BWFE), egg weight of first egg (EWFE), body weight at 300 d old (BW300), egg weight at 300 d old (EW300) and egg number by 300 d old (EN300). Genomic DNA was extracted from wing-vein blood samples using a phenol–chloroform extraction method, dissolved in ethylenediamine tetraacetic acid buffer, quantified by spectrophotometry and stored at -20 ${}^{\circ }$C for analysis.

### 2.3 DNA purity and concentration detection

The purity and concentration of each sample were detected using an ultraviolet–visible spectrophotometer. The OD260/OD280 value of each DNA sample was between 1.8 and 2.0, and the concentration was between 500 and 1,000 ng µL${}^{1-}$. These were considered acceptable. Each sample was diluted to 100 ng µL${}^{1-}$ and stored at -20 ${}^{\circ }$C for later use.

### 2.4 Primer design and polymerase chain reaction amplification

Using the *AMH* gene sequence of chickens (GenBank accession no. NC_006115.5), a pair of primers were designed using Primer v5.0 software to amplify the fragment containing exon 2 of the *AMH* gene. (Primer sequences are as follows: F: TGATATACAGCTAAACCG; R: CTTAGTCATTTCAAACACCAAGGA.) The other primer sequences are available upon request. Polymerase chain reaction (PCR) was performed in a 20 µL reaction volume, consisting of 1 µL of genomic DNA, 0.8 µL each primer; 7.4 µL dH${}_{2}$O and 10 µL 2 × Taq Master Mix for polyacrylamide gel electrophoresis (Vazyme Biotech Co., Ltd., Nanjing, China). The PCR amplification procedure was as follows: initial denaturing at 95 ${}^{\circ }$C for 5 min; 30 cycles of denaturing at 95 ${}^{\circ }$C for 30 s, annealing at 58 ${}^{\circ }$C for 30 s (a PCR gradient test indicated that the actual annealing temperature of the primer is 58 ${}^{\circ }$C) and extending at 72 ${}^{\circ }$C for 30 s; followed by a final extension at 72 ${}^{\circ }$C for 10 min. The PCR products of each genotype were sequenced by Sangon Biotech Co., Ltd. (Lou et al., 2018).

### 2.5 Polymorphism of the mutation analysis of the *AMH* exon 2

#### Gene diversity

Observed heterozygosity (H) was calculated as the proportion of total heterozygous individuals. Expected H was estimated using the following formula (Aminafshar, 2008)

1 $$H=1-\sum_{i=1}^{n}P_{i}^{2}.$$

#### Polymorphism information content (PIC)

The polymorphism information content (PIC) is an index to measure polymorphism and is especially suitable for molecular markers. When PIC > 0.5, this locus is highly polymorphic; when PIC < 0.25, this locus is slightly polymorphic. The PIC is calculated as follows:

2 $$\mathrm{PIC}=1-\left(\sum_{i=1}^{n}P_{i}^{2}\right)-\sum_{i=1}^{n-1}\sum_{j=1}^{n}2P_{i}^{2}P_{j}^{2},$$

where n is the number of alleles, $P_{i}$ is the i allele frequency and $P_{j}$ is the j allele frequency.

#### Number of effective alleles ($N_{e}$)

The number of effective alleles ($N_{e}$) is another indicator of gene purity.

In the preliminary genetic analysis, the allele number (N) and frequencies were determined by direct counting. The effective number of alleles ($N_{e}$) was calculated using the following formula (Aminafshar 2008):

3 $$N=1/\sum_{i=1}^{n}P_{i}^{2}.$$

### 2.6 Statistical analyses

The results of the DNA sequencing were analyzed by DNAMAN v5.2 and Chromas v2.31 software to determine the SNP sites in the *AMH* gene of Jinghai Yellow chickens and whether the predicted gene mutations altered the protein composition. PHASE v.2.1.1 software (Stephens and Scheet, 2005) was used to analyze the type and frequency of haplotypes as well as the correlations between genotypes and reproductive traits. The differences among the haplotypes of the *AMH* gene in Jinghai Yellow chickens were compared using least squares variance analysis with the following statistical model:

4 $$Y=\mu +G_{i}+e,$$

where Y represents the phenotypic value of the trait, μ represents the population mean, $G_{i}$ represents the fixed effects of genotype or diplotype, and e represents the random residual error. Data were listed as mean ± standard deviation (SD). The SPSS 20.0 GLM (general linear model) program was used for all statistical analyses. P<0.01 was considered extremely significant.

**Figure 1:**
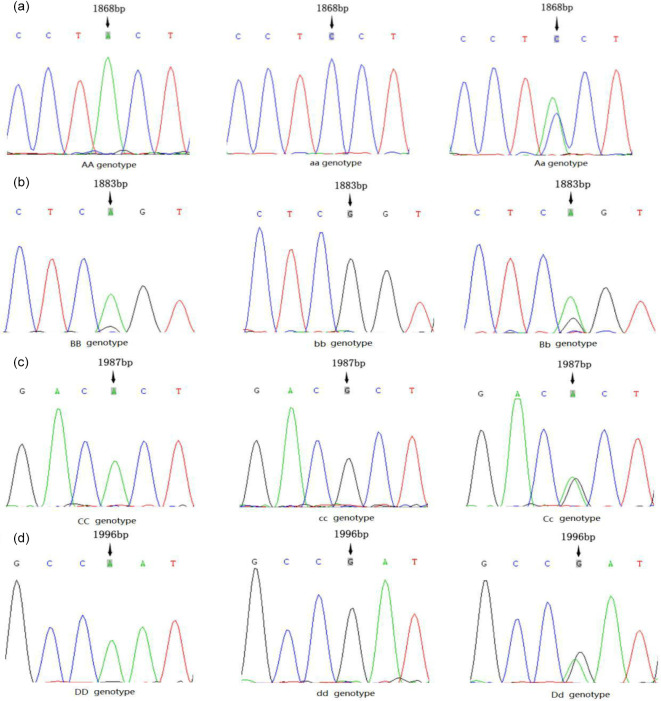
Genotype consisting of four mutation sites.

## 3 Results

### 3.1 Mutation site detection

The PCR products were sequenced, and four mutation sites were found in the second exon of the *AMH* gene; however, no polymorphism was detected in other exons in our population. As shown in Fig. 1, the three genotypes of the g.A1868C site were AA, Aa and aa; those of the g.G1883A site were BB, Bb and bb; those of the g.G1987A site were CC, Cc and cc; and those of the g.A1996G site were Dd, dd and DD. The DNAMAN analysis showed that all four SNP sites caused amino acid changes, resulting in missense mutations. The mutation of A>C at the g.A1868C site caused a change from tyrosine (Tyr) to proline (Pro). The mutation of G>A at the g.G1883A site caused a change from arginine (Arg) to serine (Ser). The mutation of G>A at the g.G1987A site caused a change from alanine (Ala) to histidine (His). The mutation of A>G at the g.A1996G site caused a change from asparagine (Asn) to arginine (Arg).

### 3.2 Genotypic and allelic frequencies of the *AMH* exon 2 mutation sites

The genotypic and allele frequency of each mutation site in the *AMH* exon 2 are shown in Table 1. The genotypic frequencies of alleles A and a at the g.A1868C site were 0.815 and 0.185, respectively. The genotypic frequencies of alleles B and b at the g.G1883A site were 0.955 and 0.045, respectively. The genotypic frequencies of alleles C and c at the g.G1987A site were 0.82 and 0.18, respectively. The genotypic frequencies of alleles D and d at the g.A1996G site were 0.43 and 0.57, respectively. As shown in Table 2, the g.G1883A locus has a low polymorphism rate, and the g.A1868C, g.G1987A and g.A1996G loci have medium polymorphism rates. The p value of the chi square test for each mutation site was less than 0.000001, which does not conform to Hardy–Weinberg equilibrium (HWE) (Table 2).

**Table 1:** Allele and genotype frequencies of the *AMH* exon 2.

Loci	Genotype frequency			Allele frequency	
gA1868C	AA	aa	Aa	A	a
	0.72	0.09	0.19	0.815	0.185
gA1883G	BB	bb	Bb	B	b
	0.94	0.03	0.03	0.955	0.045
gG1987A	CC	cc	Cc	C	c
	0.75	0.11	0.14	0.82	0.18
gA1996G	DD	dd	Dd	D	d
	0.35	0.49	0.16	0.43	0.57

**Table 2:** Polymorphism of the mutation in the *AMH* exon 2.

Loci	(H)	(PIC)	($N_{e}$)	$\chi^{2}$	p value
gA1868C	0.30	0.257	1.44	36.46	P<0.000001
gG1883A	0.09	0.08	1.09	94.36	P<0.000001
gG1987A	0.29	0.25	1.41	64.31	P<0.000001
gA1996G	0.49	0.37	1.96	112.54	P<0.000001

H represents heterozygosity, PIC represents polymorphism information content and $N_{e}$ represents effective number of alleles. PIC > 0.5 means high polymorphism. 0.25 < PIC < 0.5 means moderate polymorphism. PIC < 0.25 means a low polymorphism.

### 3.3 Correlation analysis between genotypes and reproductive performance

Results of the correlation analysis showed that only the g.A1996G locus among the four SNPs influenced the reproductive traits in the present population. There were no significant differences between the different genotypes and reproductive traits for the other loci. At the g.A1996G locus, the AFE of the dd genotype was significantly earlier than that of the DD genotype (p<0.01). Compared with the DD genotype, the hen AFE of the dd genotype was 5.73 d earlier. In addition, although there was no significant difference among genotypes regarding the number of eggs laid by 300 d old, the dd genotype laid the largest number of eggs (112.16), which indicates that dd is the beneficial genotype with respect to both the earliest egg production and the greatest number of eggs laid by 300 d.

**Table 3:** Association analysis between different genotypes of the *AMH* exon 2 and reproductive traits in Jinghai Yellow chickens (mean ± SD).

Site	Genotype	AFE (d)	BWFE (g)	EWFE (g)	BW300 (g)	EW300 (g)	EN300
gA1996G	DD	149.49 ± 11.89${}^{A}$	1677.78 ± 193.32	33.35 ± 6.19	2063.73 ± 300.86	50.71 ± 5.34	102.91 ± 27.26${}^{A}$
	Dd	147.62 ± 12.43${}^{A}$	1659.29 ± 189.41	33.69 ± 6.54	2093.45 ± 286.10	51.62 ± 3.65	100.95 ± 29.80${}^{A}$
	dd	143.76 ± 9.58${}^{B}$	1650.10 ± 175.12	33.92 ± 8.39	2032.55 ± 268.37	50.94 ± 5.17	112.16 ± 29.59${}^{B}$

AFE: age at first egg; BWFE represents the body weight at first egg, EWFE represents the egg weight of the first egg, BW300 represents the body weight at 300 d old, EW300 represents the egg weight at 300 d old and EN300 represents the egg number by 300 d old. Different capital letters in the same column show extremely significant difference (p<0.01).

**Table 4:** Haplotypes of four mutations in the *AMH* exon 2.

Haplotype	gA1868C	gG1883A	gG1987A	gA1996G	Frequency (%)
H1	A	G	G	A	44.15
H2	A	G	G	G	24.43
H3	A	G	A	A	1.36
H4	A	G	A	G	9.75
H5	A	A	A	G	1.61
H6	C	G	G	A	2.19
H7	C	G	G	G	11.54
H8	C	G	A	A	0.46
H9	C	G	A	G	4.49

### 3.4 Haplotype and diplotype analyses of each mutation site of *AMH* exon 2

Using the four SNP sites, a haplotype analysis was carried out using Phase 2 software (Table 4). Theoretically, the number of haplotypes should be 16; however, nine haplotypes were in fact found in Jinghai Yellow hens, of which H1 (A-G-G-A) and H2 (A-G-G-G) were the major haplotypes, accounting for 44.15 % and 24.43 % of the population, respectively. Based on these nine haplotypes, 17 combinations of haplotypes were obtained (Table 5). To ensure the accuracy of the analysis, genotypes representing less than 0.1 % of the population were excluded from the analysis.

### 3.5 Correlation analysis between haplotype combinations and reproductive performance of Jinghai Yellow chickens

We conducted a correlation analysis on individuals with a haplotype combination frequency greater than 5 % (Table 6). The EN300 of the H7H7 haplotype was the highest (at 113.87) followed by H2H2 (111.44), and the EN300 of the H7H7 and H2H2 haplotypes was significantly greater than that other haplotypes (p<0.01). There were no significant differences between the different haplotypes and the following reproductive traits: BWFE, EWFE, BW300 and EW300. AFE and EN300 are both important reproductive performance indicators. Therefore, H2H2 and H7H7 were determined to be the optimal haplotype combination.

**Table 5:** Diplotypes and frequencies of the four mutations in the *AMH* exon 2.

Diplotype	Frequency	Diplotype	Frequency
	(%)		(%)
H1H1	33.33	H2H4	2.85
H1H2	8.13	H2H7	4.07
H1H3	1.63	H4H4	4.07
H1H4	3.67	H4H7	2.85
H1H6	2.03	H4H5	1.63
H1H7	5.28	H4H9	1.63
H1H8	1.22	H7H7	6.10
H1H9	0.81	H9H9	2.44
H2H2	15.45		

**Table 6:** Association analysis between diplotypes in the *AMH* exon 2 and reproductive traits (means ± SD).

Diplotypes	AFE (d)	BWFE (g)	EWFE (g)	BW300 (g)	EW300 (g)	EN300
H1H1(79)	149.49 ± 11.89${}^{A}$	1677.78 ± 193.32	33.35 ± 6.19	2063.73 ± 300.86	50.71 ± 5.34	102.91 ± 27.26${}^{A}$
H1H2(20)	147.00 ± 12.61${}^{A}$	1640.75 ± 214.40	33.50 ± 6.30	2125.25 ± 343.54	51.13 ± 4.23	91.60 ± 33.21${}^{A}$
H1H7(13)	150.15 ± 9.64${}^{A}$	1679.62 ± 188.96	36.54 ± 7.47	2068.46 ± 272.15	51.88 ± 3.50	104.54 ± 29.03${}^{A}$
H2H2(36)	143.53 ± 9.49${}^{B}$	1663.06 ± 185.96	34.44 ± 8.52	2053.19 ± 242.77	50.46 ± 5.45	111.44 ± 30.69${}^{B}$
H7H7(15)	144.33 ± 10.12${}^{B}$	1619.00 ± 147.00	32.67 ± 8.21	1983.00 ± 325.82	52.09 ± 4.39	113.87 ± 27.69${}^{B}$

AFE represents the age at first egg, BWFE represents the body weight at first egg, EWFE represents the egg weight of first egg, BW300 represents the body weight at 300 d old, EW300 represents the egg weight at 300 d old and EN300 represents the egg number by 300 d old. Different capital letters in the same column denote extremely significant differences (p<0.01).

## 4 Discussion

In reproductive research, *AMH* is considered to be closely related to follicular development, both in mammals and poultry (Johnson et al., 2012). *AMH* expression varies in different stages of follicular development. In preantral follicles and small antral follicles (≤4 mm), *AMH* expression is highest, whereas in antral follicles (>8 mm), *AMH* expression is low or even not detectable (Weenen et al., 2004). In mammals, the *AMH* concentration shows a decreasing trend as the follicle diameter increases. The expression levels of *AMH* and *AMH* receptor II in buffalo small follicles (3 mm) are much higher than in large follicles (>8 mm) and are significantly negatively correlated with the estrogen concentration (Liang et al., 2016). The *AMH* gene is very important for follicular growth and maturation. Different concentrations of *AMH* injected into different hens have different effects on follicular development and progesterone secretion. It has also been shown that the higher the *AMH* concentration, the lower the ovary weight and the number of follicles in hens (Lkhagvagarav, 2019). Chen et al. (2018) detected the expression of the *AMH* gene in different grades of follicles in ducks using quantitative PCR technology. The *AMH* expression decreased gradually with follicular development. They hypothesized that *AMH* plays an important role in follicular development in ducks. The high expression level in the early developmental stage helped to maintain the undifferentiated state of granulosa cells, ensuring the normal development of follicles (Chen et al., 2018). Therefore, in animals, the level and regularity of *AMH* expression play important roles in the follicular development.

Reproductive traits are important for the economic value of chickens. Under the same feeding and management conditions, the reproductive capacities of various chickens differ. One important reason for this is the changes in base sequences and then amino acid sequences that result from gene mutations. In this experiment, four mutation sites were found in exon 2 of the *AMH* gene by sequencing the genomic DNA, and each mutation site resulted in a different genotype. Genotypes corresponding to the g.G1868C site were Aa, aa and AA; those corresponding to the g.G1883A site were Bb, bb and BB; those corresponding to the g.G1987A site were Cc, CC, and cc; and those corresponding to the g.A1996G site were Dd, dd and DD. Thus, the *AMH* gene appears to have an abundance of polymorphisms in different animal breeds that correspond with the comprehensive functions of the gene.

Haplotype analyses combining multiple loci take the interactions between non-alleles and the linkage disequilibrium before SNP loci into account, resulting in higher statistical efficacies (Horne et al., 2004). In this experiment, four mutation sites were found in exon 2 of the *AMH* gene. In theory, there should be 2${}^{4}$ haplotypes, but there were in fact nine effective haplotypes in this population. This may be related to the existence of linkage disequilibrium, the density of SNP sites and the sample size. In this experiment, the presence of four mutation sites resulted in changes in amino acids. Mutation g.A1868C resulted in a change from tyrosine to proline, mutation g.G1883A resulted in a change from arginine to serine, mutation g.G1987A resulted in a change from alanine to histidine and mutation g.A1996G resulted in a change from asparagine to arginine. Additionally, at the g.A1996G site, the age of first egg production for the dd genotype was significantly lower than that of the DD genotype (p<0.01), indicating that the dd genotype was the beneficial genotype for the age of onset. The EN300 is an important indicator of the fecundity of chickens. The dd genotype had a higher EN300 value than the other two genotypes, which indicates that the mutation at the g.A1996G site of the *AMH* gene affects the reproductive performance of Jinghai Yellow chickens, which is consistent with previous research results. Therefore, we speculate that the dd genotype can be used as a genetic marker for molecular breeding of chickens. In the diplotype analysis, there were significant differences in the EN300 and AFE values. The H1H1, H1H2 and H1H7 individuals had significantly higher AFE values than H2H2 and H7H7 individuals (p<0.01), which indicated that H2H2 and H7H7 may be a beneficial diplotype with respect to AFE. The H2H2 and H7H7 individuals had significantly higher EN300 values than the other diplotypes in hens (p<0.01), which also represented a beneficial combination. Therefore, it can be concluded that the H2H2 and H7H7 diplotype was the best combination in terms AFE and EN300. A haplotype or haplotype block analysis provides a practical way to resolve the innate problems associated with a single-marker analysis, such as high background noise, unsatisfactory correlations and obscured localization information (Daly et al., 2001). Both haplotype diversity and SNP selection based on maximum haplotype diversity are preferred (Huang et al., 2003).

## 5 Conclusions

Using a comprehensive analysis, four SNPs sites were found in the second exon of the *AMH* gene in Jinghai Yellow chickens, including the g.1996A>G mutation, which had a very significant correlation with reproductive traits. The dd genotype was determined to be optimal. Compared with haplotype combinations, the diplotype combinations showed more obvious genetic effects. H2H2 and H7H7 individuals had the optimal combination of AFE and EN300 values. In the production of Jinghai Yellow chickens, breeding the optimal genotype will help reach production goals.

## Data Availability

The datasets used and/or analyzed during the current research are available upon request.

## Code availability

PHASE is available online at (Stephens and Scheet, 2005) and can be downloaded from .
